# Exploring the Impact of Dietary EPA/DHA Supplementation on Lipid Metabolism of *Tenebrio molitor* Larvae

**DOI:** 10.3390/insects16101007

**Published:** 2025-09-28

**Authors:** Qiwei Liu, Xiangxiang Ni, Chengcheng Chen, Jingjing Xu, Enqi Pei, Aifen Yang, Mingfeng Xu, Xiu Wang, Sida Fu, Rongrong Yu

**Affiliations:** 1 College of Life and Environmental Sciences, Hangzhou Normal University, Hangzhou 311121, China; 2023112010020@stu.hznu.edu.cn (Q.L.); 2023112010006@stu.hznu.edu.cn (X.N.); 2023112010046@stu.hznu.edu.cn (C.C.); 2022210301202@stu.hznu.edu.cn (J.X.); 2024210301010@stu.hznu.edu.cn (E.P.); aifen_yang@hznu.edu.cn (A.Y.); 2 School of Advanced Materials Engineering, Jiaxing Nanhu University, Jiaxing 314001, China; 3 College of Biological and Chemical Engineering, Jiaxing University, Jiaxing 314001, China; 4 Department of Pediatrics, The First Affiliated Hospital of Wenzhou Medical University, Wenzhou 325000, China

**Keywords:** *Tenebrio molitor*, lipid metabolism, EPA, DHA, nutritional value

## Abstract

*Tenbrio molitor* (*T. molitor*) is a widely utilized feed ingredient, though it is deficient in long-chain omega-3 fatty acids, such as eicosapentaenoic acid (EPA) and docosahexaenoic acid (DHA). To address this, dietary supplements containing EPA and DHA in ethyl ester and triglyceride forms were administered to investigate the lipid metabolism and bioenhancement potential of *T. molitor*. The larvae exhibited normal growth across all treatment groups. EPA/DHA levels were significantly elevated in *T. molitor*-enriched diets, with newly identified phospholipid species including phosphatidylcholine 18:1_20:5 (PC 18:1_20:5) and phosphatidylethanolamine 18:0_20:5 (PE 18:0_20:5). KEGG pathway analysis revealed that glycerol phospholipid metabolism (ko00564), endogenous cannabinoid signaling (ko04723), and cell division (ko04148) were the core pathways that promoted phospholipid synthesis and oxidative lipid conversion (such as peroxide value-phosphatidylcholine, POV-PC). *T. molitor* activates glycerophospholipid metabolism, converting EPA/DHA into more bioavailable medium- and short-chain phospholipids, thereby enhancing its nutritional value and providing a new strategy for the development of functional foods/feeds.

## Simple Summary

*Tenebrio molitor* (*T. molitor*) larvae are rich in high-quality fats, but lack essential omega-3 fatty acids, specifically docosahexaenoic acid (DHA) and eicosapentaenoic acid (EPA). This study investigated the incorporation of fish oil-containing DHA and EPA in both ethyl ester and triglyceride-forms, into the diet of *T. molitor* larvae. The results demonstrated that the larvae effectively converted these exogenous fatty acids into more bioavailable phospholipids, significantly improving their nutritional profile. Although a high dose of fish oil slightly reduced weight gain, the larvae maintained healthy growth conditions. Furthermore, mealworms act as natural “bioconverters”, transforming poorly absorbed omega-3 compounds into more biologically accessible forms. These findings offer promising strategies for developing nutritionally enhanced foods and feeds, with important implications for human and animal health.

## 1. Introduction

*Tenebrio molitor* (TM) is rich in high-quality protein, fats, and minerals, making its nutritional value comparable to that of conventional livestock and poultry meats [1]. TM lipids are abundant in bioactive compounds, with oleic acid (36.8%) and linoleic acid (32.4%) being the major fatty acids. The ratio of polyunsaturated fatty acids (PUFAs) to saturated fatty acids (SFAs) is similar to that of vegetable oils, and its health promotion index (2.42) is ten times higher than that of common animal fats [2]. Additionally, TM oil contains significant amounts of carotenoids, tocopherols, and phenolic compounds (such as apigenin), which contribute to its strong antioxidant capacity [2]. TM exhibits remarkable digestive adaptability and efficient bioconversion ability, enabling it to thrive on low-value substrates such as bran, agricultural waste, and food processing residues [3,4]. It also shows potential in biodegrading plastics [5], highlighting its promise in value-added biotransformation. TM is already widely used as feed, which has been shown to improve meat quality in farmed species without compromising safety [6,7,8,9]. It has been approved as a novel food ingredient in the European Union [10]. Amid growing global protein demand, TM farming offers a resource-efficient and environmentally sustainable alternative and is considered a viable solution for future food systems [11,12,13,14]. However, the low content of omega-3 PUFAs (<0.5%) in TM lipids limits its application as a functional food ingredient. This study aims to explore the metabolic mechanisms of TM in converting dietary eicosapentaenoic acid (EPA) and docosahexaenoic acid (DHA) through biofortification strategies, with the goal of developing TM-based products with enhanced nutritional value.

DHA and EPA, omega-3 fatty acids derived mainly from marine sources, exhibit diverse health benefits including anti-inflammatory, cardioprotective, and neuroprotective effects [15,16,17,18,19]. However, their bioavailability is limited by inherent structural characteristics—such as esterification forms (ethyl ester or glyceride)—which impede intestinal absorption and reduce utilization efficiency [20,21]. Although advanced delivery systems such as phospholipid liposomes can improve bioavailability [22], conventional formulations remain limited by high cost, instability, and complex processing. Given the robust digestive and metabolic capabilities of TM, this study incorporated EPA and DHA into the diet to investigate their influence on the lipid metabolism of TM. We aimed to elucidate how dietary EPA and DHA enrich the fatty acid profile of TM and reveal the molecular mechanisms underlying lipid metabolic remodeling, thereby demonstrating the potential for nutrient enhancement in TM.

In summary, given the natural limitation of omega-3 fatty acids in TM, this study aims to utilize the known nutritional benefits of EPA and DHA along with TM′s lipid digestive plasticity to address its lipid nutritional gaps. By investigating the impact of dietary EPA and DHA supplementation on lipid metabolism and overall nutritional composition in TM, this work seeks to establish a theoretical foundation for developing TM-based products with optimized fatty acid profiles and provide empirical support for enhancing the physiological benefits and bioavailability of EPA and DHA.

## 2. Materials and Methods

### 2.1. Rearing and Treatment

*Tenebrio molitor* larvae were purchased from the Shandong Yellow Mealworm Breeding Base (Shandong, China). Dried bran was served as the basal feed, to which different types of EPA and DHA were supplemented: ethyl ester EPA (EE-EPA), ethyl ester DHA (ED-DHA), and triglyceride DHA (TG-DHA) (each with purity > 90%, and purchased from the Skuny Bioscience Co. Ltd., Chengdu, China). The three forms of EPA and DHA were supplemented into the basal diet at concentrations of 2.5%, 5%, and 10% (*w*/*w*), respectively.

TM larvae were randomly divided into four groups: a control group (CK), an EE-EPA group, an ED-DHA group, and a TG-DHA group. Each group was further supplemented with the corresponding lipids at three concentration gradients (2.5%, 5%, and 10%, *w*/*w*), with three biological replicates per concentration. The larvae were reared in ventilated plastic containers (5.5 × 5.5 × 4.5 cm) with 50 individuals per container, under controlled conditions of 25 °C, 70% relative humidity, and continuous darkness. Feeding was performed every two days, and the body weight of the TM larvae was regularly recorded. The rearing period lasted 4–5 weeks, until the larvae reached maturity at the 7th to 8th instar stage. Prior to formal treatment, the larvae were subjected to a 3-day starvation period to avoid potential interference from residual feed with subsequent analytical results. During the rearing period, larvae, feed, frass, and exuviae were regularly separated by sieving. Finally, all TM samples were rapidly frozen and stored at −20 °C for further analysis.

### 2.2. Gas Chromatography (GC)

#### 2.2.1. Lipid Extraction

Lipids were extracted using the Folch method [23]. Frozen TM larvae were pulverized and homogenized using a chloroform–methanol solution (2:1, *v/v*) in a 1:3 (*w/v*) ratio for 3 min. The mixture was subjected to low-temperature ultrasonic extraction for 20 min, followed by centrifugation (10 °C, 8500 rpm) for 15 min to collect the supernatant. A volume of distilled water was added to the supernatant, and after thorough mixing, the solution was centrifuged again under the same conditions. The lower lipid-containing phase was collected, evaporated under nitrogen gas, and dried to obtain high-purity lipid extracts. The samples were dissolved in 0.5 mol/L methanolic potassium hydroxide solution, filtered through a 0.22-μm membrane, and stored at 4 °C for further analysis.

#### 2.2.2. Detection by GC

The samples were analyzed using an Agilent 7890B GC system (Agilent Tech., Santa Clara, CA, USA). The GC conditions were set as follows: Needle rinse solvent was n-hexane; the sample injection volume of 10 μL and an injection purge flow rate of 6000 μL/min. The column (30 m × 250 μm × 0.2 μm) was operated at a flow rate of 1.5 mL/min, a pressure of 16.531 psi, an average linear velocity of 36.078 cm/s, and a retention time of 1.3859 min. The injector heater was set to 280 °C, with hydrogen (H_2_) flow at 30 mL/min, air flow at 300 mL/min, and makeup gas (N_2_) flow at 30 mL/min. The total run time was 33.667 min. Before each injection, the syringe was rinsed with n-hexane and the sample solution to prevent cross-contamination.

### 2.3. Non-Targeted Metabolomics

#### 2.3.1. Lipid Metabolite Extraction

Lipids were extracted using 1 mL of a pre-cooled extraction solvent (isopropanol/acetonitrile/water = 2:1:1, IPA:ACN:H_2_O = 2:1:1, *v/v/v*). A 100 mg samples were extracted, vortesed for 1 min, and then incubated at room temperature for 10 min. The extract was then stored at −20 °C overnight. After centrifugation at 4000× *g* for 20 min, the supernatant was transferred to a new 96-well plate. Samples were stored at −80 °C prior to Liquid chromatography–mass spectrometry (LC-MS) analysis, which was performed using an ACQUITY UPLC system (Waters, Milford, MA, USA) and a TripleTOF 6600 mass spectrometer (SCIEX, Framingham, MA, USA). At the same time, each extraction solution was added to 10 μL to prepare mixed QC samples.

#### 2.3.2. Liquid Phase Parameter Description

Analyses were performed using an LC-MS system. Chromatographic separations were performed using the ACQUITY UPLC system (Waters, Milford, MA, USA). A Reverse-phase separation was performed using a Kinetex UPLC C18 column (100 mm × 2.1 mm, 100 Å, Phenomenex, Macclesfield, UK). Column temperature was maintained at 55 °C. The flow rate was 0.3 mL/min. The mobile phase consisted of solvent A (ACN:H_2_O = 6:4, 0.1% formic acid) and solvent B (IPA:ACN = 9:1, 0.1% formic acid). The gradient elution conditions were set as follows: 0–0.4 min, 30% B; 0.4–1 min, 30–45% B; 1–3 min, 45–60% B; 3.5–5 min, 60–75% B; 5–7 min, 75–90% B; 7–8.5 min, 90–100% B; 8.5–8.6 min, 100% B; 8.6–8.61 min, 100–30% B; 8.61–10 min, 30% B.

#### 2.3.3. Description of Mass Spectrum Parameters

Metabolites eluting from the column were detected using a high-resolution tandem mass spectrometer, the TripleTOF 6600 system (SCIEX, Framingham, MA, USA). The instrument was operated in both positive and negative ion modes. The following parameters were applied: curtain gas at 30 psi, ion source gas 1 at 60 psi, ion source gas 2 at 60 psi, and the interface heater temperature was set to 650 °C. The ion spray voltage floating was set to 5000 V for positive ion mode and to −4500 V for negative ion mode. Mass spectrometry data were acquired in information-dependent acquisition (IDA) mode. The TOF mass range was set from 60 to 1200 Da. Survey scans were acquired over 150 ms, and up to 12 product ion scans were triggered when the intensity exceeded 100 counts per second (cps) with a +1 charge state. The total cycle time was fixed at 0.56 s. For each scan, four time bins were summed at a pulser frequency of 11 kHz using a 40 GHz multichannel time-to-digital converter (TDC) detector equipped with four-anode channel detection. Dynamic exclusion was set to 4 s. During acquisition, mass accuracy was recalibrated every 20 samples. To evaluate the stability of the LC-MS system throughout the entire sequence, a quality control (QC) sample (pooled from all samples) was analyzed after every 10 experimental injections.

### 2.4. Statistics and Analysis

#### 2.4.1. Basic Data

The growth data of TM were processed using Origin software (Origin 2018, OriginLab Corporation, Northampton, MA, USA), and statistical analyses were performed with IBM SPSS software (IBM SPSS Statistics 26, IBM, Armonk, NY, USA). Data were analyzed using either Student’s *t*-test or two-way analysis of variance (ANOVA), followed by Tukey′s post hoc test. When *p* < 0.05 (the threshold between groups). A *p*-value of less than 0.05 was considered statistically significant.

#### 2.4.2. Comparison and Analysis of Non-Targeted Metabolomics

Raw mass spectrometry data were preprocessed using XCMS software version 4.0.2 (Scripps Res. Inst., La Jolla, CA, USA) for peak picking, peak grouping, retention time correction, secondary alignment, and isotope/adduct annotation. Data processing was performed via the MATLAB-based XCMS toolbox (University of Innsbruck, Innsbruck, Austria). Each ion was identified based on its retention time (RT) and *m*/*z* data. The intensity of each peak was recorded to generate a three-dimensional matrix containing any specified peak index (retention time-*m*/*z*), sample name (observation), and ion intensity information (variable).

The metabolites were labeled using online kyoto encyclopedia of genes and genomes (KEGG) lipid metabolites and pathways strategy (LIPID MAPS) and human metabolome databases (HMDB), and the precise molecular mass data (*m*/*z*) of the samples were matched with the data in the database. The metabolite identification was verified using the in-house database.

Multivariate statistical analysis, such as cluster heat map, principal component analysis (PCA), hierarchical cluster analysis (HCA) and KEGG pathway enrichment, were used to compare the differences in fatty acid metabolic profiles among all groups, and to screen the significant changes in lipid metabolites.

## 3. Results

### 3.1. TM Growth

**Figure 1:**
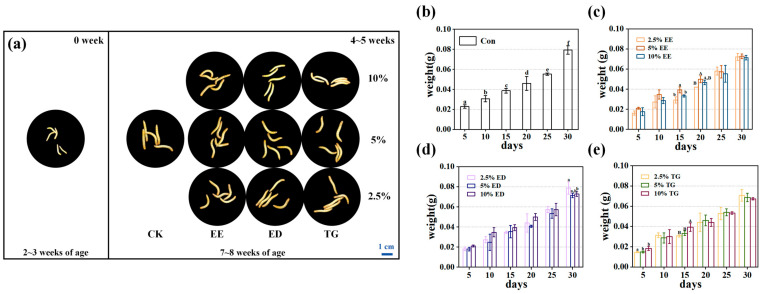
Changes in body weight of TM over the rearing period (0–5 weeks): (**a**) Morphological changes in TM across groups (N = 5). CK: Control; EE: EE-EPA; ED: EE-DHA; TG: TG-DHA. (**b**) The body weight changes in the control group. (**c**) The body weight changes in the EE-EPA group. (**d**) The body weight changes in the ED-DHA group. (**e**) The body weight changes in the TG-DHA group. Among them, the different symbols in the annotations of groups a–f and A,B indicate that there are significant differences among different groups within this group (at the same time) (*p* < 0.05). Data are expressed as mean ± standard deviation (SD).

The TM larvae were reared according to the experimental protocol, and all groups exhibited normal growth throughout the study period (Figure 1). By weeks 4–5, the TM had reached a significantly larger size and displayed a noticeably darker coloration compared to their initial state. No significant differences in body size or coloration were observed between the control group and any of the experimental groups (EE-EPA, ED-DHA, or TG-DHA). Furthermore, within each experimental group, varying feeding concentrations did not lead to significant differences in these morphological characteristics. Overall, the TM demonstrated healthy growth across all conditions (Figure 1a).

In the control group, the body weight of TM increased with prolonged rearing time, and significant differences (*p* < 0.05) were observed between successive time points (Figure 1b). In TM groups fed with different concentrations of EE-EPA, ED-DHA, and TG-DHA, body weight also increased over the rearing period. Notably, significant differences in body weight were observed within the EE-EPA treatment group on day 15 (*p* < 0.05) and day 20. In the ED-DHA treatment, significant intergroup differences were detected on day 30 (*p* < 0.05). In the TG-DHA treatment, the body weight of TM reared at the 2.5% concentration showed a significant difference compared to those at the other two concentrations on day 5 (*p* < 0.05), while on day 15, the body weight under the 10% concentration differed significantly from the other two groups (*p* < 0.05) (Figure 1b–e). However, the body weight of TM in all three experimental groups was slightly lower than that in the control group. The addition of EPA/DHA may affect the feeding behavior of TM on bran. Previous studies have shown that omega-3 fatty acids not only exert beneficial effects against chronic diseases, but may also contribute to reductions in body weight, waist circumference, and body mass index (BMI) when consumed in appropriate amounts [24]. This may explain why the body weight of TM in the experimental groups was slightly lower than that in the control group. In addition, the highest body weight was observed in the TG-DHA and ED-DHA groups at a concentration of 2.5%, while the EE-EPA group achieved optimal growth at a concentration of 5% (Figure 1c–e).

In conclusion, supplementation with appropriate concentrations of TG-DHA, ED-DHA, and EE-EPA did not adversely affect the normal growth of TM. Therefore, we performed a comprehensive analysis of lipid metabolism and nutritional composition in each group of TM.

### 3.2. The Lipid Metabolites of TM Were Preliminarily Detected by GC

**Figure 2:**
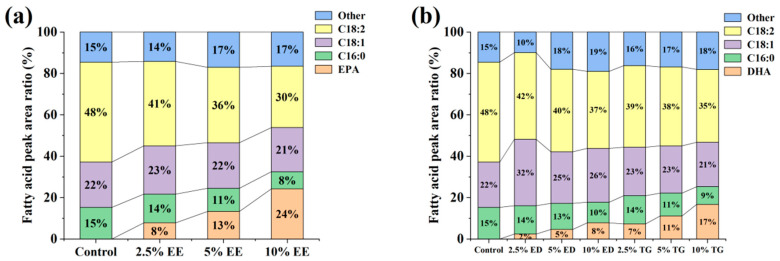
The changes in the peak area occupied by some lipid metabolite components of TM under GC detection: (**a**) The changes in lipid components in TM after feeding different concentrations of EE-EPA. (**b**) Changes in lipid components in TM after feeding with different concentrations of ED-DHA and TG-DHA.

To distinguish the effects of EE-EPA, ED-DHA and TG-DHA at different concentrations on the lipid components of TM, we conducted an initial analysis of the lipid composition in each group of TM using GC (Figure 2). The main fatty acids contained in TM are oleic acid (C18:1), linoleic acid (C18:2) and palmitic acid (C16:0) [25]. In the control group, the peak area percentages were approximately 48% for C18:2, 22% for C18:1, and 15% for C16:0. The relative peak area of EPA increased proportionally with the concentration of EE-EPA, whereas those of C18:2 and C16:0 decreased with increasing EE-EPA concentration (Figure 2a). Similarly, the peak area ratio of DHA was directly proportional to the concentrations of TG-DHA and ED-DHA, while C18:2 and C16:0 were inversely proportional to the concentrations of TG-DHA and ED-DHA (Figure 2b). These results suggest that the intake of exogenous EPA/DHA by TM may influence the endogenous levels of C18:2 and C16:0 in TM.

Based on the experimental results of this section, we have gained a preliminary understanding of the changing trends in the lipid composition of TM under different feeding conditions. We therefore proceeded to conduct a more detailed investigation of TM lipid metabolism. Furthermore, since appropriate concentrations of EPA/DHA did not inhibit normal growth of TM, and since the 10% supplementation groups of TG-DHA, EE-DHA, and EE-EPA showed the highest potential for EPA/DHA enrichment, we selected the 10% supplementation groups for further analysis.

### 3.3. Non-Targeted Metabolomics

#### 3.3.1. TG-DHA, ED-DHA and EE-EPA Affected the Lipid Metabolism of TM

**Figure 3:**
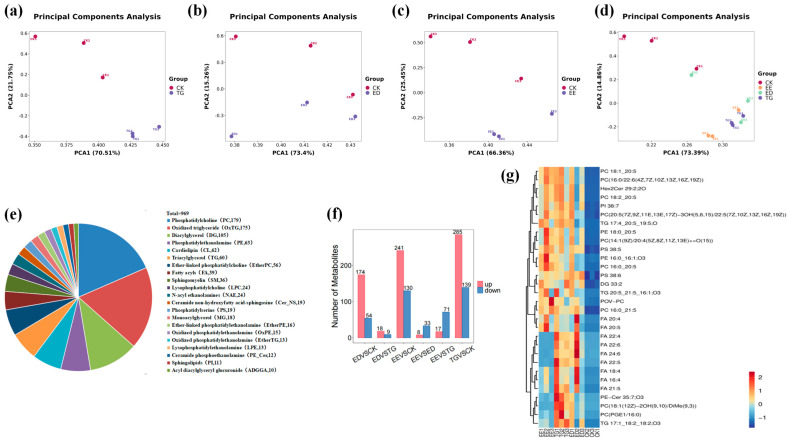
TG-DHA (TG), ED-DHA (ED) and EE-EPA (EE) affected the lipid metabolites of TM: (**a**) PCA of the TM control group in comparison to the TG-DHA group (n = 3). (**b**) PCA of the TMcontrol group in comparison to the ED-DHA group (n = 3). (**c**) PCA of the TM control group in comparison to the EE-EPA group (n = 3). (**d**) PCA of the TM control group in comparison to the three experimental groups (n = 3). (**e**) Quantity distribution of major lipid categories in all samples detected by HPLC-MS/MS. (**f**) The quantities of lipid differential metabolites in the six groups of CK and ED, CK and EE, CK and TG, ED and EE, ED and TG, and TG and EE (n = 3). (**g**) Heat maps of lipid metabolites of CK, ED, EE and TG.

To further understand the effects of EE-EPA, ED-DHA and TG-DHA at a concentration of 10% on the lipid metabolites of TM, we adopted the lipid metabolite detection method based on high performance liquid chromatography-mass spectrometry (HPLC-MS) to analyze the four groups of samples. PCA of lipid metabolism revealed significant differences between the experimental groups (TG-DHA, ED-DHA, EE-EPA) and the control group (PC1 + PC2 > 80%) (Figure 3a–c). This clear separation indicates that the TM lipid metabolism pathways and products were altered by the intervention. Furthermore, the proximity between the EE-EPA group and the TG-DHA group was relatively high, indicating that their metabolic profiles were similar. This cluster was slightly different from the ED-DHA group and significantly different from the control group, suggesting distinct metabolic pathways and products among these groups (PC1 + PC2 > 80%) (Figure 3d). A total of 969 main lipid subclasses were obtained from each group of samples through MS/MS detection and analysis, including 179 phosphatidylcholines (PC), 105 oxidized triglycerides (OxTG), 63 phosphatidylethanolamines (PE), 60 triacylglycerols (TG), and 39 fatty acyl groups (FA) and other lipid classes (Figure 3e). Among them, compared with the CK group, the types of lipid metabolites in all three intervention groups increased significantly. Compared with the CK group, TG-DHA increased 285 metabolites, ED-DHA increased 174 metabolites compared with the CK group, and EE-EPA increased 241 metabolites compared with the CK group (Figure 3f). Among these lipid metabolites, compared with the CK group, 19 lipid metabolites were upregulated in all three experimental groups, while an additional 11 metabolites were upregulated in the TG-DHA group. Compared with the three experimental groups, the types of lipid metabolites in the CK group were all down-regulated (Figure 3g).

To sum up, all three Omega-3 fatty acids have significantly affected the lipid metabolites of TM. Based on the results of this experiment, taking some major lipid metabolites as examples, we further explored the effects of TG-DHA, ED-DHA and EE-EPA on the lipid metabolism of TM.

#### 3.3.2. Analysis of the Changes of 9 Major Lipid Metabolites in Each Group

The main lipid metabolites obtained from each group of TM samples by HPLC-MS/MS detection mainly include several types such as PC, FA, TG and PE. Taking nine major lipid metabolites as examples (Table 1), we investigated the potential effects of TG-DHA, ED-DHA, and EE-EPA on the lipid metabolism of TM.

**Figure 4:**
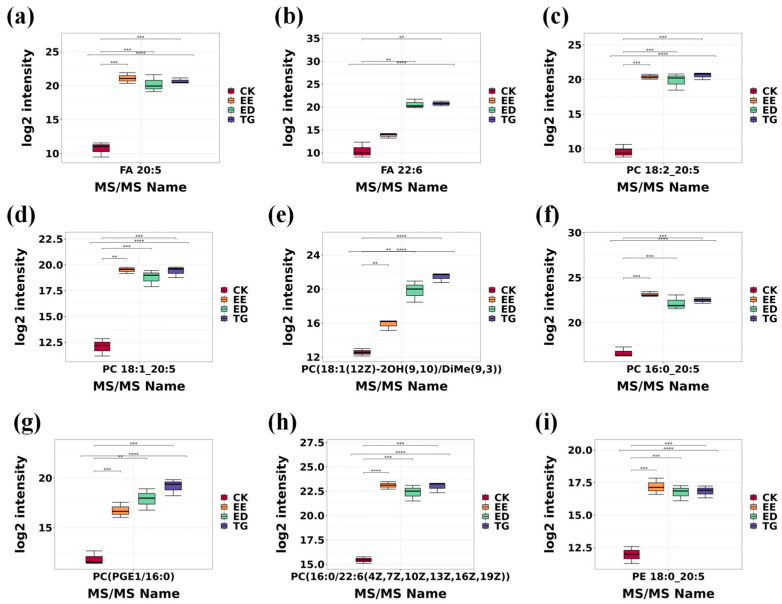
Box plots of the changes in nine major lipid metabolites in the CK group and the three intervention groups of EE-EPA, ED-DHA, and TG-DHA: (**a**) FA 20:5. (**b**) FA 22:6. (**c**) PC 18:2_20:5. (**d**) PC 18:1_20:5. (**e**) PC (18:1(12Z)-2OH(9,10)/DiMe(9,3)). (**f**) PC 16:0_20:5. (**g**) PC (PGE1/16:0). (**h**) PC (16:0/22:6(4Z,7Z,10Z,13Z,16Z,19Z)). (**i**) PE 18:0_20:5. IQR: The third quartile (Q3) to the first quartile (Q1) in the box, which is used to illustrate the dispersion and concentration of the data in this group. The smaller the IQR, the more concentrated the data; conversely, the smaller the IQR, the more dispersed the data. Among them, ** indicates very significant (*p* < 0.01), *** indicates extremely significant (*p* < 0.001), **** indicates an extremely strict significance level (*p* < 0.0001).

A comparative analysis of these nine lipid metabolites was conducted in the form of a box plot. The IQR values of each group were all relatively small (0 < IQR < 5), and in the obtained dataset, they were analyzable (Figure 4). The detection intensity of FA 20:5 (EPA) in the TG-DHA, ED-DHA and EE-EPA groups was significantly higher than that in the CK group, and there were extremely significant differences between the three groups and the CK group, respectively (*p* < 0.001). Among them, the detection intensity of the EE-EPA group was the highest, followed by the TG-DHA group and the ED-DHA group. Based on the detection results, it is inferred that the intervention with EE-EPA likely resulted in the accumulation of EPA in TM, leading to an increase in its concentration, which aligns with the experimental expectations. However, the detection intensity of EPA in the ED-DHA and TG-DHA groups also showed enhancement, which might be related to the transformation of the TM lipid metabolism pathway. Further exploration of this reason is needed (Figure 4a). In addition, the detection intensity of FA 22:6 (DHA) in the TG-DHA and ED-DHA groups was significantly higher than that in the CK group, while it was slightly higher in the EE-EPA group. This finding indicates that interventions with TG-DHA and ED-DHA likely promoted the accumulation of DHA in TM, leading to increased concentration levels, which is consistent with the experimental hypothesis. Among them, there were very significant differences between the ED-DHA group and the CK group, as well as between the TG-DHA group and the CK group (*p* < 0.01) (Figure 4b). It is notable that the interventions of TG-DHA, ED-DHA and EE-EPA all increased the detection intensity of PC in the lipids contained in TM, and were significantly higher than that in the CK group. For the metabolite PC 18:1 20:5, the difference between CK and EE-EPA was very significant (*p* < 0.01). The differences between CK and ED-DHA, CK and TG-DHA were extremely significant (*p* < 0.001). For the metabolite PC (18:1(12Z)-2OH(9,10)/DiMe(9,3)), the differences between CK and EE-EPA, CK and ED-DHA were very significant (*p* < 0.01), and the difference between CK and TG-DHA was extremely significant (*p* < 0.001). For the metabolite PC 16:0_20:5, the differences between CK and EE-EPA, CK and ED-DHA, and CK and TG-DHA were extremely significant (*p* < 0.001). For the metabolite PC (PGE1/16:0), the differences between CK and EE-EPA, CK and TG-DHA were extremely significant (*p* < 0.001). The difference between CK and ED-DHA was very significant (*p* < 0.01). For the metabolite PC (16:0/22:6(4Z,7Z,10Z,13Z,16Z,19Z)), the differences between CK and EE-EPA were extremely strictly significant (*p* < 0.0001). The differences between CK and ED-DHA, CK and TG-DHA were extremely significant (*p* < 0.001). Based on the above results, we infer that these three fatty acids may promote the synthesis of this class of phospholipids in TM, potentially through pathways associated with phospholipid biosynthesis or degradation metabolism in TM (Figure 4c–h). PE 18:0_20:5 is composed of SFA 18:0 (stearic acid) and PUFA 20:5, and plays an important role in the structure and function of cell membranes as well as the regulation of mitochondrial functions [26,27]. The interventions of TG-DHA, ED-DHA and EE-EPA all significantly increased the detection intensity of this complex fatty acid. Among them, the detection intensity of the EE-EPA group was the highest, followed by TG-DHA. In addition, there were extremely significant differences between CK and EE-EPA, CK and ED-DHA, and CK and TG-DHA (*p* < 0.001) (Figure 4i). Based on the alterations observed in these nine major lipid metabolites, we investigated the underlying mechanisms driving these changes in TM lipid metabolism using KEGG enrichment analysis and metabolic pathway network mapping.

#### 3.3.3. Regulatory Analysis of EPA/DHA Intervention on the TM Lipid Metabolism Pathway

**Figure 5:**
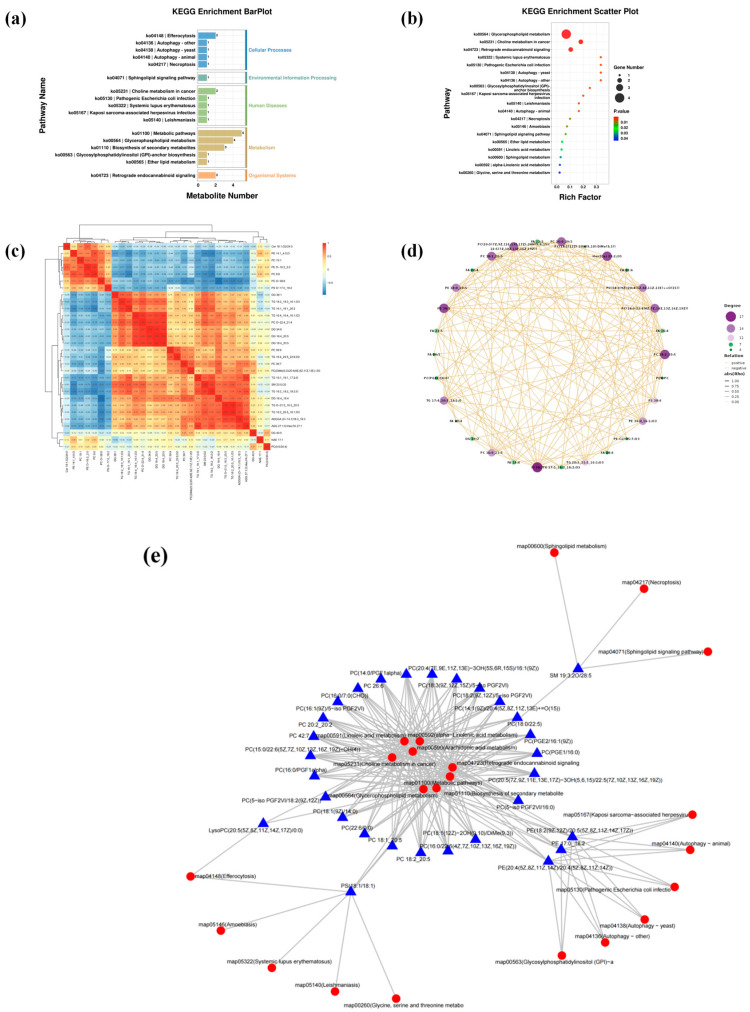
Regulatory analysis of the TM lipid metabolism pathway under the intervention of EPA/DHA (TG-DHA vs. ED-DHA vs EE-EPA vs. CK, n = 4). (**a**) Bar graph of KEGG enrichment analysis of TM lipid metabolism pathways. (**b**) Scatter plot of KEGG enrichment analysis of TM lipid metabolism pathways. (**c**) Thermal map correlation analysis of TM lipid metabolites. (**d**) Correlation network map analysis of TM lipid metabolites. (**e**) Network map analysis of TM lipid metabolites-metabolic pathways, with red circles representing lipid metabolic pathways and blue triangles representing lipid metabolites.

The above results indicated that the detection intensities of key lipid metabolites—including EPA, DHA, PC metabolites, and PE(18:0/20:5)—were significantly increased in the TG-DHA, ED-DHA, and EE-EPA experimental groups. Therefore, we speculated that TM might possess a specific capacity for absorbing and metabolizing EPA and DHA from the feed, potentially activating related lipid metabolic pathways. To further investigate the regulatory mechanism, we adopted KEGG pathway enrichment analysis and Spearman correlation analysis to systematically explore the potential signaling pathways related to differential lipid metabolites among TG-DHA, ED-DHA, EE-EPA and CK groups. KEGG pathway enrichment analysis revealed that differential lipid metabolites were mainly enriched in key biological pathways such as metabolism, cellular processes, and biological systems (Figure 5a). Among them, the effects of the four metabolic pathways, namely Ko 00564 (glycerophospholipid metabolism), Ko 04723 (reverse endogenous cannabinoid signaling), Ko 04148 (efferocytosis), and Ko 05231 (choline metabolism in cancer), were the most significant, and the amount of metabolites obtained was the largest (Figure 5b). In the Ko 00564 metabolic pathway, the existing choline can generally be used for phosphorylation, and then cytidine diphosphate glycerol (CDP) is linked as a carrier to combine with glycerol diesters and thereby form PC. PE is formed by the combination of acetamide phosphate generated through acetamide with cytosine nucleoside triphosphate (CTP) to form CDP-ethanolamine, which is then combined with diglycerol ester [28,29]. The Ko 05231 metabolic pathway is the main pathway of choline metabolism and can provide choline as a raw material for the synthesis of PC for the Ko 00564 signaling pathway [30]. The endogenous cannabinoids in the metabolic pathway of Ko 04723 originate from membrane phospholipids such as PE and PC, and their metabolism is directly related to Ko 00564 [31]. Exposure to apoptotic cell membrane phospholipids (such as PE/PC) activates the Ko 04148 signaling pathway, and therefore, this pathway is also directly related to glycerophospholipid metabolism. Furthermore, sphingolipids in Ko 04071 (sphingolipid signaling pathway) are also involved in the regulation of cell burial (Figure 5a,b) [32]. FA 20:5 and FA 22:6 might be the residues absorbed or present in the body after TM is ingested into the feed. Several major lipid metabolites in the three experimental groups were formed on the basis of the above metabolic pathways by combining lipids such as C18:0, C18:1, C18:2 and C16:0 contained in TM itself with lipid molecules such as C 20:5 and C 22:6 of fatty acids TG, ED and EE. Based on these results, it can be inferred that TM may possess a strong capacity to digest and break down EPA and DHA, which are not easily absorbed by the human body, suggesting its potential as a green and highly nutritious alternative food source. Furthermore, intervention with these three lipids significantly enriched the fatty acid profile of TM and enhanced its nutritional value.

To clarify the relationship between lipid metabolites and metabolic pathways, we constructed Spearman correlation heat maps and network maps using 969 lipid metabolites with significant differences and 22 lipid metabolic signaling pathways (Figure 5c–e). It is notable that some highly significant metabolites, phosphatidylethanolaminelyso 18:2, were positively correlated with PC(18:2/0:0) and LysoPC(18:2(9Z,12Z)/0:0), respectively. NAE 18:2, NaE 18:1 and NaE 16:0 were positively correlated with each other, and LPC 20:5 was positively correlated with PC(0:0/20:4). Furthermore, among the most significant lipid metabolism pathways, the Ko 00564, Ko 05231 and Ko 04723 pathways were the production cores of lipid metabolites of TM. The pathway Ko 04148 is mainly associated with the generation of metabolites PS(18:1/18:1) and LysoPC(20:5(5Z,8Z11Z,14Z,17Z)/0:0).

## 4. Discussion

TM is rich in high-quality fats such as C18:1 and C18:2. It can be used as feed to improve meat quality and nutritional value in grass carp, and as a dietary protein source for pigs without adverse metabolic effects [33]. Additionally, TM thrives on agricultural waste and shows considerable potential for bioplastic degradation. However, its near absence of omega-3 fatty acids limits its nutritional applications. Omega-3 fatty acids such as EPA and DHA are effective in treating chronic inflammatory and neurodegenerative diseases, yet their bioavailability is limited due to poor intestinal absorption [24,34,35]. Most therapeutic omega-3 formulations require emulsification to enhance absorption [36,37]. Based on the lipid composition and metabolic profile of TM, this study investigated the effects of dietary supplementation with EPA and DHA on lipid metabolism in TM.

Among the three concentration gradients of 2.5%, 5%, and 10%, the contents of C22:6 and C20:5 in TM of the three groups of fatty acid experimental groups at a concentration of 10% (g/g) were the highest. It is worth noting that although the body weight of TM in each group increased significantly and grew normally, the body weight of TM at a concentration of 10% was relatively low. Two potential reasons are speculated as follows: 1) the high-concentration EPA/DHA exhibited higher viscosity, and its odor potentially interfered with TM, leading to reduced feed intake. 2) EPA/DHA showed the effects of ameliorating obesity and promoting fat metabolism, which may lead to lower body weight in normally growing TM. The detection intensity of HPLC-MS /MS can reflect the content of each fatty acid from the side. It was found that the richness of TM lipid metabolites increased after the intervention. In addition to the significant increase in the contents of FA22:6 and FA20:5, the contents of major lipid metabolites such as PC 18:2_20:5, PC 18:1_20:5, PC 16:0_20:5 and PE 18:0_20:5 all increased significantly (*p* < 0.05). However, it should be noted that when comparing the increased metabolites among the CK, EE-EPA, TG-DHA, and ED-DHA groups via HPLC-MS, the detection intensities (relative concentrations) of FA 20:5 and the metabolite PC(16:0/22:6(4Z,7Z,10Z,13Z,19Z)) were remarkably similar across the three experimental groups (Figure 4a,h). Based on the experimental methodology and results, we speculate that this phenomenon may be related to lipid metabolic pathways in TM, such as Ko04723 [31], or to characteristics inherent to the detection instrumentation. Further investigation using internal standards for absolute quantification of lipid metabolites could help elucidate this observation. The formation of novel lipid complexes is attributed to the enzymatic synthesis of phospholipids and triglycerides that incorporate both the ingested fatty acids (C22:6, C20:5) and TM’s endogenous fatty acids (such as C18:0, C18:1, C18:2, C16:0) into their structure. We adopted the methods of KEGG enrichment analysis, Spearman correlation analysis network diagram and clustering heat map to clarify the main metabolic pathways involved in these reactions. Analysis revealed that under the intervention of fish oil, Ko 00564-glycerophospholipid metabolism, Ko 04723-reverse endogenous cannabinoid signal transduction, Ko 04148-burial effect, and Ko 05231-choline metabolism were the main metabolic pathways of TM (*p* < 0.05). In the current analysis, phosphatidylcholines (PC, 179 species), oxidized triglycerides (OxTG, 105 species), and phosphatidylethanolamines (PE, 63 species) were identified as the predominant classes of lipid metabolites (*p* < 0.05). In cellular burial metabolism, exposure to membrane phospholipids activates the glycerophospholipid metabolic pathway. Glycerol phospholipid metabolism utilizes the existing choline in choline metabolism for phosphorylation, and then connects to CDP as a carrier to combine with glycerol diesters to form PC [38]. Phosphorylation of acetamide combines with CTP to form CDP-ethanolamine, which then combines with diglycerol ester to form PE [39]. In addition, the oxidative stress or lipid peroxidation reaction of TM can lead to the oxidation of fatty acid chains in TG to form OxTG. The glycerol diester generated by the oxidative degradation of OxTG can participate in the phospholipid metabolic pathways of PC and PE. Apoptotic cells release oxidized phospholipids (such as POV-PC), a lipid that participates in the cytoplasmic metabolic pathway and is subsequently eliminated [40]. However, it is worth noting that the presence of POV-PC was detected in the lipid metabolites of all three intervention groups (*p* < 0.05). We speculate that this phenomenon may be attributed to interactions between the cellular burial metabolic pathway of TM and other major metabolic pathways, leading to the accumulation of POV-PC as a predominant intermediate metabolite form in TM. However, the specific underlying mechanisms require further exploration through other signaling pathways.

In conclusion, dietary supplementation with EPA and DHA significantly enriched the fatty acid profile of TM and enhanced its nutritional value without compromising normal growth. Furthermore, we also observed an enhanced lipid metabolic profile in TM larvae (Figure 5), particularly a significant upregulation of phospholipids. This reflects, to some extent, strategies employed in synthetic delivery systems—such as phycocyanin-cationic starch complexes—which are designed to improve UV stability and bioavailability of sensitive nutraceuticals [41]. Theoretically, these findings also suggest that *Tenebrio molitor* could function as a natural bioreactor for producing bioavailable omega-3 phospholipid fatty acids. Future studies may focus on the biological functions and mechanistic roles of the identified lipid metabolites. This exploration provides valuable data and a theoretical foundation for the utilization of TM in fields such as food science, chemistry, and pharmaceuticals.

## 5. Conclusions

In this study, dietary supplementation with EPA and DHA enriched the fatty acid profile of TM, revealing the molecular mechanism of lipid metabolism remodeling in TM and its potential for nutritional fortification. The experimental results showed that EPA and DHA supplementation significantly increased the content of omega-3 fatty acids in TM and promoted the synthesis of novel phospholipid complexes such as PC 18:1_20:5 and PE 18:0_20:5. Metabolomics analysis showed that the types of lipid metabolites in the intervention group increased significantly, including 179 types of PC, 105 types of OxTG and 63 types of PE. KEGG enrichment analysis further revealed that glycerol phospholipid metabolism, reverse endogenous cannabinoid signaling, cytoburism and choline metabolism are the core pathways for the conversion of TM lipid metabolism to EPA/DHA. These pathways work in synergy to convert long-chain omega-3 fatty acids into metabolic complexes in the form of PC, PE, and others that have been absorbed [42,43].

Although the weight gain of TM in the 10% EE-EPA, ED-DHA, and TG-DHA groups was lower than that of the control group, all groups maintained normal growth and development, indicating that EPA/DHA supplementation did not compromise TM health. More importantly, TM has demonstrated its potential as a bioreactor capable of converting poorly absorbable EPA and DHA into more bioactive forms, such as PC and PE, providing an innovative strategy for developing novel omega-3 fortified foods [41,42,43]. This study not only offers a theoretical basis for nutrient enhancement in TM but also expands its potential applications in functional foods and medical fields. Future studies should prioritize the use of internal standard methods for absolute quantification of these lipid metabolites, followed by systematic assessment of their biological activities.

## Figures and Tables

**Table 1:** Major lipid metabolites.

s	*m*/*z*	MS/MS Name	Formula	Class	*p* Value	*q* Value
1	301.22	FA 20:5	C_20_H_30_O_2_	FA	0.00	0.00
2	327.23	FA 22:6	C_22_H_32_O_2_	FA	0.00	0.00
3	848.54	PC 18:2_20:5	C_46_H_78_NO_8_P	PC	0.00	0.00
4	850.55	PC 18:1_20:5	C_46_H_80_NO_8_P	PC	0.00	0.00
5	830.55	PC (18:1(12Z)-2OH(9,10)/DiMe(9,3))	C_44_H_80_NO_11_P	PC	0.00	0.00
6	780.53	PC 16:0_20:5	C_44_H_78_NO_8_P	PC	0.00	0.00
7	832.565	PC(PGE1/16:0)	C_44_H_82_NO_11_P	PC	0.00	0.00
8	806.55	PC (16:0/22:6(4Z,7Z,10Z,13Z,16Z,19Z))	C_46_H_80_NO_8_P	PC	0.00	0.00
9	764.51	PE 18:0_20:5	C_43_H_76_NO_8_P	PE	0.00	0.00

## Data Availability

The original data presented in the study are openly available in https://www.scidb.cn/en/s/EnyE3m (accessed on 23 September 2025).

## Abbreviations

The following abbreviations are used in this manuscript:

Abbreviations	Full name
CDP	Cytidine diphosphate glycerol
CK	Control group
CTP	Cytosine nucleoside triphosphate
DHA	Docosahexaenoic acid
ED-DHA	Ethyl ester docosahexaenoic acid
EE-EPA	Ethyl ester eicosapentaenoic acid
EPA	Eicosapentaenoic acid
FA	Fatty acyl groups
FAO	Food and Agriculture Organization of the United Nations
GC	Gas chromatography
GDP	Cytidine diphosphate glycerol
GTP	Cytosine nucleoside triphosphate
HCA	Hierarchical cluster analysis
HMDB	Human metabolome databases
HPLC-MS	High performance liquid chromatography-mass spectrometry
HPLC-MS/MS	High Performance Liquid Chromatography-Tandem Mass Spectrometry
IDA	Information-dependent acquisition
KEGG	Kyoto encyclopedia of genes and genomes
LC-MS	Liquid chromatography-mass spectrometry
LIPID MAPS	Lipid metabolites and pathways strategy
OxTG	Oxidized triglycerides
PC	Phosphatidylcholines
PCA	Principal component analysis
PE	Phosphatidylethanolamines
POV-PC	Peroxide value-phosphatidylcholine
PUFAs	Polyunsaturated fatty acids
QC	Quality control
SFAs	Saturated fatty acids
TDC	Time-to-digital converter
TG	Triacylglycerols
TG-DHA	Triglyceride docosahexaenoic acid
TM	*Tenebrio molitor*
